# Reinventing henna: Enzyme‐catalysed colour release from stabilized *Lawsonia inermis* L. extracts

**DOI:** 10.1111/ics.70029

**Published:** 2025-10-20

**Authors:** Nele Dallmann, Volkmar Vill, Fabian Straske

**Affiliations:** ^1^ Henkel AG & Co. KGaA Hamburg Germany; ^2^ Department of Organic Chemistry University of Hamburg Hamburg Germany

**Keywords:** colour cosmetics, formulation/stability, hair treatment, lawsone, plant‐based hair colouration, β‐Glucosidase

## Abstract

**Objective:**

Improvement of application, performance, colour predictability and stability of plant‐based hair colourants derived from *Lawsonia inermis* L. by investigating the release mechanism of lawsone during extraction and optimizing processing conditions to prevent its premature formation. A two‐component system was developed, consisting of naturally occurring lawsone precursors (hennosides) in one formulation and a catalytic agent (β‐glucosidase) in another.

**Methods:**

Henna raw material was extracted using maceration, Soxhlet, and industrial‐scale methods with various solvents. Extracts were analysed for yield and content of lawsone, hennosides, polyphenols, metals and reducing sugars. HPLC quantified lawsone and hennosides; reducing sugars and polyphenols were measured using dinitrosalicylic acid and Folin–Ciocalteu assays, respectively. Yak hair was dyed with different formulations and evaluated for colour outcome and wash fastness using the CIELab colour system.

**Results:**

Formulations containing pre‐released lawsone were unstable and ineffective after 4 weeks. In contrast, gel formulations containing hennosides without β‐glucosidase remained stable for over 8 weeks and produced consistent colour when combined with the enzyme before application. Ethanolic extraction effectively yielded hennoside‐rich extracts without lawsone formation. Aqueous extraction at boiling point also produced hennoside‐rich extracts. Soxhlet extraction resulted in β‐glucosidase activity remaining in the starting material. The two‐component gel formulation demonstrated comparable wash fastness and colour intensity to the conventional paste, with improved ease and speed of application and rinsing.

**Conclusion:**

Optimizing henna extract processing and formulation design enabled the development of a more user‐friendly, effective and stable plant‐based hair dye, supporting broader consumer adoption.

## Graphical abstract

The instability and reduced dyeing performance of a cosmetic formulation containing 2‐hydroxy‐1,4‐naphthoquinone (lawsone), derived from *Lawsonia inermis* L., is demonstrated in comparison to a novel two‐component system. This system comprises one formulation with naturally occurring lawsone precursors and a second containing a specific hydrolase. Mixing both components shortly before application enables a stable and reproducible colour outcome, even after prolonged storage.

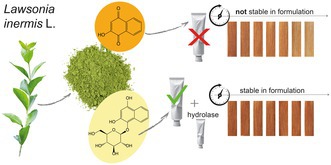

## RESUME

**Objectif:**

Amélioration de l'application, de la performance, de la prévisibilité des couleurs et de la stabilité des colorants pour cheveux d'origine végétale dérivés de *Lawsonia inermis* L. en étudiant le mécanisme de libération de la lawsone pendant l'extraction et en optimisant les conditions de traitement pour prévenir sa formation prématurée. Un système à deux composants a été développé, constitué de précurseurs de la lawsone (hennosides) naturellement présents dans une formulation et d'un agent catalytique (β‐glucosidase) dans une autre.

**Méthodes:**

La matière première du henné a été extraite à l'aide de méthodes de macération, Soxhlet, et à l'échelle industrielle avec divers solvants. Les extraits ont été analysés en termes de rendement et de teneur en lawsone, hennosides, polyphénols, métaux et sucres réducteurs. La chromatographie en phase liquide à haute performance (HPLC) a quantifié la lawsone et les hennosides; les sucres réducteurs et les polyphénols ont été mesurés à l'aide de dosages de l'acide dinitrosalicylique et de Folin–Ciocalteu, respectivement. Des poils de yak ont été teintés avec différentes formulations et évalués pour déterminer le résultat en termes de couleur et la résistance au lavage à l'aide du système de couleur CIELab.

**Résultats:**

Les formulations contenant de la lawsone pré‐libérée étaient instables et inefficaces après 4 semaines. En revanche, les formulations en gel contenant des hennosides sans β‐glucosidase sont restées stables pendant plus de 8 semaines et ont produit une couleur constante lorsqu'elles étaient associées à l'enzyme avant l'application. L'extraction éthanolique a effectivement produit des extraits riches en hennosides sans formation de lawsone. L'extraction aqueuse au point d'ébullition a également produit des extraits riches en hennosides. L'extraction Soxhlet a permis de conserver l'activité β‐glucosidase dans le matériau de départ. La formulation en gel à deux composants a démontré une résistance au lavage et une intensité de couleur comparables à celles de la pâte conventionnelle, avec une facilité et une vitesse d'application et de rinçage améliorées.

**Conclusion:**

L'optimisation de la conception du traitement et de la formulation de l'extrait de henné a permis de développer une teinture capillaire à base de plantes plus conviviale, plus efficace et plus stable favorisant une adoption plus large par les consommateurs.

## INTRODUCTION

The plant‐based hair dye market, albeit still niche, is forecasted to grow from $2.1 billion in 2023 to $4.3 billion by 2032 [1]. Increasing consumer awareness of the origin and biodegradability of cosmetic products [2], along with rising incidences of allergic reactions to ingredients such as para‐phenylenediamine (PPD) found in conventional oxidative hair dyes [3], is expected to be a key driver of market growth. Traditionally, plant‐based hair colourations are available as finely ground plant powder that is prepared freshly by the consumer before application [4]. The preparation, application and aftercare can be tedious and messy [5] as the paste is often prepared with hot water requiring cooling time, followed by application times from 30 min to several hours, followed by a difficult removal by rinsing dried‐up plant material from the hair.

Even though plants like henna (*Lawsonia inermis* L.) and indigo (*Indigofera tinctoria* L.) are widely known and used for hair dyeing purposes [6], there is little innovation regarding liquid cosmetic formulations containing and marketing exclusively plant‐based, natural hair colourations in the EU. Aspects like the mentioned micro‐market status [1] of plant‐based hair colouration, as well as strict safety and application guidelines [7] are factors that can easily discourage the cosmetic industry from investing and further exploring plants as a viable alternative for oxidative hair dyes. But with more consumers interested in natural and biodegradable cosmetics [2], the main focus for product developers and investors should be to make plant‐based hair colouration more accessible.

The main aspect of reducing application time and messiness by incorporating *Lawsonia inermis* L. extracts into formulations is further explored in this work. The colour release mechanism of henna is investigated and strategically used to improve the consumer's experience. The characteristic orange colouration of henna, attributed to lawsone (2‐hydroxy‐1,4‐naphthoquinone), represents both its primary functional attribute and its limiting factor in application. Lawsone is not only poorly soluble in water and UV‐sensitive but requires specific extraction conditions [8] to be concentrated enough to allow for further processing such as hair colouration. Extracts suitable for hair colour formulation not only contain dyes and their precursors—such as lawsone or indican—but other secondary metabolites like polyphenols as well [8]. Polyphenols are known to bind to proteins, for example keratins, via hydrogen bonding and hydrophobic interactions [9, 10] and can form complexes with other polyphenolic and quinonoid structures as well as several transition metals like Fe^2+^, Cu^2+^ and Zn^2+^ [11, 12, 13]. These complexes are often colourful and able to influence the overall colour outcome. While those interactions are a valuable tool for the reinvention of plant‐based colouration, they are also difficult to prevent and control—especially in formulation, complicating the development of colouration which gives reliable and consistent colour results.

This work specifically highlights the advantages of using the naturally occurring lawsone precursors, hennosides, in formulation rather than lawsone itself. Hennosides are monoglycosidic, reduced precursors of lawsone [6], characterized by their high water solubility and stability within plant leaves, thanks to their glucose moiety. These properties make them highly promising for use in water‐based, plant‐derived colouration formulations.

## MATERIALS AND METHODS

### Chemicals

The reference standard for lawsone (Lawsone, 2‐Hydroxy‐p‐naphthoquinone, 99%, CAS: 83‐72‐7, Lot: A0435140) was purchased from Thermo Scientific (Geel, Belgium). HPLC‐grade solvents were purchased from VWR International GmbH (Darmstadt, Germany). Ultra‐pure water (UPW) was freshly provided through an Arium Pro Ultra‐Pure Water System by Sartorious AG (Göttingen, Germany). Water purity was measured by conductivity, with a limiting resistivity of 18.2 MΩ cm. Analytical reagent grade absolute ethanol ≥99.8% (CAS: 64‐17‐5) was purchased from Fisher Scientific (Loughborough, UK). Diethyl ether ≥99% (CAS: 60‐29‐7) was purchased from VWR International GmbH (Darmstadt, Germany). Formic Acid ≥90% for analytical purposes (CAS: 64‐18‐6) was purchased from Merck KGaA (Darmstadt, Germany). For the gel applications, 100% bio‐based Caprylyl Glycol, A‐Leen® 8 (CAS: 1117‐86‐8; Lot: 2/21) was kindly provided by Minafin SPRL (Mont‐Saint Guibert, Belgium). Xanthan Gum (CAS: 11138‐66‐2, Lot: 2E7233K) and Diutan Gum (CAS: 125005‐87‐0, Lot: 9K1222W) were kindly provided by Tate & Lyle PLC (London, United Kingdom). Citric acid, 99+% (CAS: 77‐92‐9) and Sodium Benzoate, 99+% (CAS: 532‐32‐1) were purchased from Thermo Scientific (Geel, Belgium). A liquid enzyme fermentation product containing 5–10% w/w β‐glucosidase of fungal origin used for hair dyeing experiments was kindly provided by AB Enzymes GmbH (Darmstadt, Germany). For quantification of total polyphenolic content (TPC), Folin–Ciocalteu reagent was purchased from Merck KGaA (Darmstadt, Germany), gallic acid (CAS: 149‐91‐7), used as a standard, was acquired from Sigma‐Aldrich (St. Louis, MO, USA), and sodium carbonate was purchased from VWR International GmbH (Darmstadt, Germany). Quantification of reducing sugars in all plant extracts was achieved by using technical grade sodium potassium tartrate tetrahydrate (CAS: 6381‐59‐5) from VWR International GmbH (Darmstadt, Germany), D‐glucose, anhydrous for biochemistry (CAS: 50‐99‐7) from Merck KGaA (Darmstadt, Germany) and 3,5‐dinitrosalicylic acid, 98% (CAS: 609‐99‐4) purchased from Thermo Scientific (Geel, Belgium).

### Plant materials

50 kg Moroccan henna (*Lawsonia inermis* L.) crushed leaves (Lot 21‐3 MARO, Nov 2021) were purchased through Croda GmbH (Nettetal, Germany). The raw material has a particle size of 10–18 mesh and a moisture content <10%.

### Extraction of *Lawsonia inermis* L. raw material

#### Aqueous extraction to obtain lawsone‐rich extract

Moroccan raw material (100 g) was extracted with 2.2 L of ultra‐pure water at 40°C for 2 h under constant stirring. The insoluble plant material was centrifuged at room temperature (RT, 23°C ± 2°C) and 4347× *g* for 30 min (Centrifuge 5910 Ri, S‐4xUniversal, Eppendorf, Germany). The extract was decanted, filtrated through quantitative filter paper, 434, 2–3 μm, VWR International GmbH, dried under vacuum, and stored under light exclusion until further usage. The extraction was repeated three times. The lawsone‐rich extracts (LE) were analysed for lawsone content via HPLC and combined for further dyeing experiments.

#### Ethanolic Soxhlet extraction targeting hennosides

The production of a hennoside‐rich extract (ESE) was completed by extracting 100 g of Moroccan henna raw material with 1 L of 96% ethanol via Soxhlet extraction over 25 cycles and 24 h, at 60°C and approx. 220 mbar. A 500 mL Soxhlet apparatus was filled with a glass extraction sleeve containing the raw material as well as 150 g of glass beads (⌀ 3 mm, VWR International GmbH, Darmstadt, Germany). To prove reproducibility, the extraction was performed four times. The liquid extracts were filtered (quantitative filter paper, 434, 2–3 μm, VWR International GmbH), dried under vacuum and stored under light exclusion until further usage. The extracts were analysed for hennoside content via HPLC and combined for further colouring experiments.

#### Aqueous extraction targeting hennosides

To prepare an aqueous heat‐denatured extract (HD) without enzyme activity, 20 g of raw material were suspended in 500 mL of boiling ultra‐pure water. The mixture was stirred on a magnetic stirrer for 5 min before cooling on ice to RT. The suspension was filtrated (quantitative filter paper, 434, 5–10 μm, VWR International GmbH), and the raw material was extracted a second time with 500 mL of boiling ultra‐pure water. After the second filtration, the solutions were combined and concentrated under vacuum to a total volume of 50 mL. The extract was frozen to −80°C and lyophilized at 1 mbar for 4 days (Alpha 1–4 LSCbasic, Martin Christ Gefriertrocknungsanlagen GmbH, Osterode am Harz, Germany) until fully dried. The extraction was executed three times to prove reproducibility. Extracts were stored under vacuum and light exclusion until further usage. After HPLC quantification of hennoside content, the extracts were combined for further colouring experiments.

#### Industrial‐scale maceration targeting hennosides

Henna leaf extraction at industrial scale was done by Indfrag Biosciences Private Limited (Bengaluru, India). A pilot plant extraction of Indian henna raw material was realized by repeated maceration of 50 kg material with 85% ethanol with a ratio of 1:10 raw material to solvent. 50 kg was extracted at 60°C for 2 h. The liquid extract was combined and concentrated. The viscous extract was then combined with water and filtrated over a high‐bed filter bed. The clear liquid was concentrated again to a thick paste and combined with 95% acetone with a ratio of 1:10. The mixture was stirred at RT for 1 h. The insoluble residue was extracted a second time with acetone. The acetone‐soluble extract was collected and dried to a powder. The dried powder was resuspended in water (1:10) and passed through polymeric resin. The extract obtained was dried completely and stored under vacuum until further usage.

### Hair material

Hair material used for all experiments was purchased from Kerling Haarfabrik GmbH (Backnang, Germany). The untreated, white, selected yak hair strands are 11.8 cm long, including the plastic bonding. Free hair fibre material is approx. 7.5 cm long and weighs 0.7 g per strand.

#### Preparation of hair material before colouration

All strands were prepared by treating them to a standardized cleansing protocol. The strands were dampened with tap water while combing under a constant water flow of 25 mL s^−1^. Afterwards, they were placed in 20 mL strand^−1^ of an aqueous 12.5% w/w Sodium Laureth Sulphate (SLES) solution adjusted to pH 4.5 (HCl, 10%) for 30 min at RT. The hair strands were rinsed under running water (25 mL s^−1^) while combing 10 times and until no surfactant residue was visible. The material was air dried overnight and stored under light exclusion until further usage.

### Hair colouration preparation and dyeing protocols

#### Gel formulation application

250 g of gel preparations (Table 1) were prepared by firstly dissolving the extracts in demineralized water. Caprylyl glycol, preservative and pH regulator were added into the solution and stirred with a stirring unit by IKA, RW 20, dissolver disk Ø 5 cm, (Staufen, Germany) until yielding a smooth solution. The thickeners were added slowly while stirring (600 rpm). The gels were left to stir for 1 h (2000 rpm); water lost during the stirring process was compensated before the final gels were stored in amber glass, airtight at 5°C over the span of 8 weeks.

**TABLE 1:** Gel formulation constituents for hair dyeing gels containing a lawsone‐rich extract (LE) and an ethanolic extract (EE).

Ingredient	LE gel formulation (% w/w)	EE gel formulation (% w/w)
Demineralized water	78.7	92.2
Lawsone‐rich extract (LE)	18.9	—
Ethanolic henna extract (EE)	—	1.4
Xanthan Gum	1.0	1.0
Diutan Gum	0.5	0.5
Caprylyl Glycol	0.5	0.5
Sodium Benzoate	0.3	0.3
Citric acid	0.1	0.1

The hair dyeing protocols for strands dyed with LE and EE gels varied slightly. For LE gel colourations, 5 g of gel per hair strand was applied, at room temperature (RT, 23°C ± 2°C), left for 30 min, rinsed under running water (25 mL s^−1^, 20°C ± 2°C) and blow‐dried (80°C ± 5°C, distance of 10 cm) before colour assessment. The EE gel preparation was prepared by combining 5 g of gel per strand with 50 μL of β‐glucosidase preparation 15 min before application. Total dyeing time was 30 min. The strands were rinsed and dried according to LE protocol. Five hair strands each were dyed directly after gel preparation, and on day 1, 7, 14, 28 and 56 after gel preparation.

#### Paste application

Preparation of henna paste was done according to the SCCS opinion on *Lawsonia inermis* L. [7]. 400 g of paste was prepared by mixing 100 g of Moroccan raw material with 300 g hot demineralized water (80°C). The paste was stored over 8 weeks at 5°C under light exclusion. 5 g of paste per strand (*n* = 5) were used to dye yak hair directly after paste preparation, and on day 1, 7, 14, 28 and 56. The total application time was 30 min, the strands were rinsed and dried according to the protocol for gel application (Gel formulation application).

#### Colouration in suspensions and solutions

Hair colourations in solution or suspension were prepared similarly. The dye mixtures were prepared in beakers. Extracts and/or plant powders were weighed out in % w/v. Per strand, 25 mL of demineralized water was combined with the required amount of plant powder or extract. The mixtures were prepared shortly before dyeing the hair strands. For applications requiring β‐glucosidase, 50 μL of liquid β‐glucosidase preparation per strand was added into the solution or suspension and left to incubate under stirring for 15 min at RT (23°C ± 2°C). For dyeing mixtures using the residue plant powder after ethanolic Soxhlet extraction (EER) as a catalyst for lawsone release, the plant powder was combined with extract and deionized water and left to incubate under stirring at RT for 15 min. Hair strands (*n* = 3) were added into the mixture and dyed for 30 min under constant stirring. The hair strands were rinsed and dried according to protocol (Gel formulation application).

#### Assessment of washing fastness

To investigate the colourfastness, dyed hair strands were washed manually. Three strands per colouration were treated with a commercially available shampoo (Schauma 7 Herbs, Schwarzkopf, pH 4.5 ± 0.2, 12% ± 0.5% SLES). The process was divided into washing cycles; a maximum of four cycles consisting of six washes each was performed, resulting in a maximum of 24 hair washes. One hair wash consisted of a 30 s pre‐rinse of the test strands under running water (25 mL s^−1^, 20°C ± 2°C) and the manual application of 0.5 g shampoo per strand in circular motions. This motion was repeated ten times from the bonded part to the tip, followed by rinsing for 30 s while combing. After each cycle (6 washes), the strands were blow‐dried (80°C ± 5°C, distance of 10 cm) and measured spectrophotometrically for their L*, a* and b* values as well as their ∆*E* values compared to the unwashed strands.

#### Colour determination with CIELab

The L*, a* and b* values of the CIELab colour space [14] were used to assess the colour change and colouration after dyeing as well as washing the strands. The values were determined using the Spectraflash 600 X (Catalogue no.: 1200‐1405, serial no.: 8633) from Datacolor, Applied Colour Systems, Inc. (Basel, Switzerland). Colorimetric data were recorded in DCI colour software. Each strand was measured four times with a 90° turn after each measurement. The mean value of all four recordings was used for colour assessment and further calculations. Difference in colours between strands was assessed by Δ*E* value (Equation 1) [14].

(1) $$∆E=\sqrt{\left({\left(∆L^{*}\right)}^{2}+{\left(∆a^{*}\right)}^{2}+{\left(∆b^{*}\right)}^{2}\right)}$$

### Analysis of *Lawsonia inermis* L. extracts

#### Quantification of hennoside and lawsone by HPLC

For the general characterization of all extracts as well as the quantification of hennoside (THNG) and lawsone contents, the Knauer AZURA® Analytical HPLC set up with Autosampler AS 6.1L (Knauer GmbH, Berlin, Germany), a column oven (column thermostat AZURA® CT 2.1, Knauer GmbH, Berlin, Germany) and the analytical column (Eurospher II 100‐5 C18, 250 × 4.6 mm, 5 μm, 100 Å, Knauer GmbH, Berlin, Germany) was used. With a constant flow rate of 1 mL min^−1^ ultra‐pure water with 0.1% formic acid (A) and acetonitrile (B) was used as eluents. Compounds were detected by AZURA Detector DAD 2.1L (Knauer GmbH, Berlin, Germany). Collected data were analysed with ClarityChrom®9 (Knauer GmbH, Berlin, Germany). Methodology and quantification were done according to recently published findings by our working group [15]. Isolated hennoside A, 1,2,4‐trihydroxynaphthalene‐1‐O‐glucoside, was used as a reference standard for the quantification of all monoglycosidic hennosides [15].

#### Quantification of total polyphenolic content by UV–Vis

The total polyphenolic content (TPC) was measured by the colour change through the formation of blue complexes between polyphenols in the extracts and Folin–Ciocalteu (FC) reagent. Gallic acid aliquots between 10 and 100 μg mL^−1^ were prepared. One mL of each dilution was mixed with 5 mL of FC reagent (0.019–0.021% w/w). After 6 min, the mixture was combined with 4 mL of an aqueous sodium carbonate solution (7.5% w/w) and left to sit for 1 h. Absorption at 765 nm before and after complex formation was measured for the standard solutions with the UV–Vis spectrophotometer Lambda 365+ (PerkinElmer, Massachusetts, United States). Quantification of TPC for all extracts was determined using the calibration curve (Equation 2).

(2) $$\begin{array}{c} \text{absorbance}\;\left(\mathrm{AU}\right)=0.114\;\left(\mathrm{AU}\;\mathrm{mL}\;{\mathrm{\mu g}}^{-1}\right)\\ \;\times \mathrm{TPC}\;\left(\mathrm{\mu g}\;{\mathrm{mL}}^{-1}\right)+0.0154\;\left(\mathrm{AU}\right) \end{array}$$

Extract samples were prepared by dissolving 10 to 25 mg in a water–ethanol solution (9:1 v/v). Complex formation and subsequent measurements were done according to the protocol used for the gallic acid standards. TPC as gallic acid equivalent for all extracts was determined in triplicates as % w/w of extracts.

#### Quantification of reducing sugars via UV–Vis

The total concentration of reducing sugars (RSC) in the extracts was measured by using the 3,5‐dinitrosalicylic acid (DNS) assay according to Khatri et al. [16]. A fresh DNS reagent was prepared by combining 30 g of sodium potassium tartrate tetrahydrate with 1 g of 3,5‐dinitrosalicylic acid dissolved in 80 mL of 0.5 N NaOH at 45°C, and adjusting the final volume to 100 mL with UPW after cooling the solution down to RT (23°C ± 2°C). Standard glucose solutions ranging from 0 to 1000 μg mL^−1^ were prepared. To 1 mL of standard solution, 2 mL of DNS reagent was added. The mixtures were incubated for 5 min at 95°C in the ThermoMixerC at 850 rpm (Eppendorf, Hamburg, Germany). After cooling on ice to RT, the samples were diluted with the addition of 7 mL UPW; absorbance was measured at 540 nm against a reagent blank. A standard calibration curve (Equation 3) was generated and used to determine the reducing sugar content in the unknown extracts.

(3) $$\begin{array}{c} \text{absorbance}\;\left(\mathrm{AU}\right)=0.0005\;\left(\mathrm{AU}\;\mathrm{mL}\;{\mathrm{\mu g}}^{-1}\right)\\ \;\times \mathrm{RSC}\;\left(\mathrm{\mu g}\;{\mathrm{mL}}^{-1}\right)+0.0052\;\left(\mathrm{AU}\right) \end{array}$$

Extracts were dissolved ranging from 800 to 1500 μg mL^−1^ depending on water solubility, expected sugar content and blank absorbance at 540 nm. Representative of each extraction, combined extracts were investigated using three solutions of different concentrations. Final RSC as D‐glucose equivalent was determined in % w/w of extract. Additionally, for a semi‐quantitative determination of the glucose content in extract solution, QUANTOFIX® Glucose test strips (Macherey‐Nagel, Düren, Germany) were used.

#### Quantification of iron in extracts using ICP‐OES

Inductively Coupled Plasma–Optical Emission Spectrometry (ICP‐OES) was employed to determine the concentrations of iron (Fe) in all plant extracts used for hair dyeing experiments. The plant extracts were subjected to microwave digestion at 250°C (START‐1500 T‐280, MLS GmbH, Leutkirch, Germany) using 5 mL of NHO_3_. The samples were diluted to a final volume of 15 mL with deionized water and measured either undiluted or after appropriate dilution with an ICP‐OES spectrometer for elemental analysis (ARCOS, SPECTRO Analytical Instruments GmbH, Kleve, Germany).

### Statistical analysis of dye outcome and washing fastness

Statistical analysis of dye outcome, colour change through washing fastness as well as lawsone content in solution was carried out with IBM SPSS Statistics version 29.0 (IBM Corp.). Normal distribution of the data was assumed. Results are presented as mean values ± standard deviation (SD).

### Artificial intelligence‐generated content

During the writing process of the Results and Discussion of this research article, ChatGPT (4.0) was sporadically used to exclusively refine the language in a scientific context.

## RESULTS AND DISCUSSION

### Stability and performance of henna extracts in gel

The main challenges for product developers besides regulatory guidelines are the extracts' stability and performance in formulation. The most straightforward approach to developing a plant‐based henna colouration involves formulating a lawsone‐rich henna extract into a gel or cream application. This cuts out the time of lawsone development that has to be considered when using *Lawsonia inermis* L. as a fresh paste. The raw material is extracted beforehand, encouraging the enzymatic lawsone release. The water‐soluble extract is then implemented into a formulation, resulting in an easy application with decreased waste and application time. Considering the maximum amount of lawsone defined as safe by the SCCS [7], the amount of extract can be adjusted to not surpass 0.35% w/w of available lawsone in the final dye mixture. The opinion on *Lawsonia inermis* L. clearly states that solely the application as a paste using ground‐up henna leaves is covered by the mentioned safety assessment [7] though complying with the reference limitation of lawsone is a critical aspect for the reinvention of plant‐based hair colouration without endangering the consumers.

Kavepour et al. [8] have determined the optimum extraction conditions at laboratory scale to be maceration of raw material for 2 h at 40°C with a ratio of distilled water to plant material of 100 mL mg^−1^. They obtained 1.85% w/w of lawsone relative to the raw material [8]. At an industrial scale, a compromise must be made between the raw material‐to‐solvent ratio and the maximum lawsone yield. Aqueous extraction of 20 g Moroccan raw material at 40°C for 2 h with a henna‐to‐solvent ratio of 45.45 mL mg^−1^ (LE) already yielded significantly less lawsone with 0.5 ± 0.1% w/w (Table 2). The material used by Kavepour et al. [8] and the henna used in this work are of different origins and likely exhibit different hennoside and therefore lawsone contents. Regardless, Kavepour et al. showed that a ratio of 10 mL mg^−1^ with otherwise identical conditions yielded just 0.14% w/w of lawsone, emphasizing that higher solvent‐to‐henna ratios yield more lawsone overall [8].

**TABLE 2:** Extract yield (% w/w), THNG (% w/w) of raw material, Lawsone (% w/w) of raw material, THNG (% w/w) of extract and Lawsone (% w/w), reducing sugars (RSC) (% w/w of extract), total phenolic content (TPC) (% w/w of extract), Iron (Fe) and Copper (Cu) amounts in ppm of extract of aqueous lawsone‐rich extracts (LE, *n* = 3) from Moroccan raw material, ethanolic Soxhlet extracts with 96% ethanol (ESE, *n* = 4) from Moroccan raw material, heat‐denatured aqueous extracts (HD, *n* = 3) of Moroccan raw material and an ethanolic extract (EE) produced at industrial scale with 85% ethanol from Indian henna raw material.

Extract	Extract yield (% w/w)	THNG (% w/w) of raw material	Lawsone (% w/w) of raw material	THNG (% w/w) of extract	Lawsone (% w/w) of extract	RSC (% w/w) of extract	TPC (% w/w) of extract	Fe (ppm) of extract
LE	31.7 ± 1.0	—	0.5 ± 0.1	—	1.9 ± 0.2	21.1 ± 3.1	6.5 ± 0.07	25
ESE	31.7 ± 0.7	5.2 ± 0.9	<0.05	16.4 ± 2.8	0.2 ± 0.1	18.7 ± 1.3	10.7 ± 0.49	<5
HD	36.9 ± 0.7	3.5 ± 0.9	<0.05	9.6 ± 2.4	0.2 ± 0.1	13.1 ± 0.3	9.9 ± 0.80	28
EE	4.0	1.2	<0.05	27.6	0.3	22.3	22.7	34

*Note*: Data are presented as the average and standard deviation.

**FIGURE 1:**
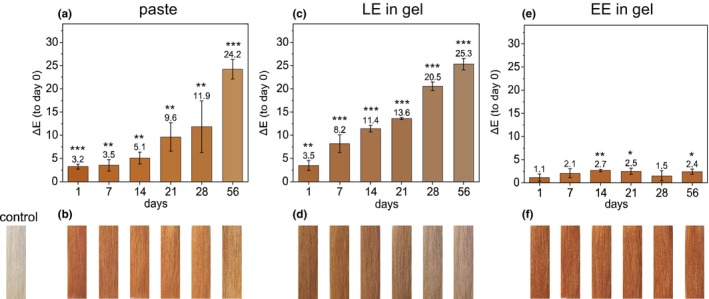
(a) Mean ± SD of ∆*E* values for hair strands dyed with henna paste (1:3 raw material to water, 5 g per strand) over 8 weeks, compared to day 0 (*n* = 5); (b) corresponding images of strands dyed on days 1–56, aligned with (a); (c) Mean ± SD of ∆*E* for strands dyed with lawsone‐rich extract (LE) in gel (5 g per strand) over 8 weeks vs. day 0 (*n* = 5); (d) images of strands dyed with LE gel on days 1–56, aligned with (c); (e) Mean ± SD of ∆*E* for strands dyed with ethanolic extract (EE) in gel (5 g gel + 50 μL β‐glucosidase per strand) over 8 weeks vs. day 0 (*n* = 5); (f) corresponding images for EE gel on days 1–56, aligned with (e). Column colours reflect CIELab values converted to RGB; significant differences vs. day 0 are marked: *p < 0.05, **p < 0.01, ***p < 0.001.

By removing the preparation time of a henna paste as well as the waiting time before application necessary for lawsone release, one could simplify the usage of *Lawsonia inermis* L. as a hair dye for the consumer. This can be achieved by either preparing a paste with ground‐up henna leaves, weight volume of 1:3, leaves to water [7] or by formulating a gel containing aqueous henna extract exhibiting the same amount of lawsone deemed safe for the paste application (Table 1, LE gel formulation). Both treatments can be prepared beforehand, packaged and stored until usage. For this work, the paste was not combined with oils or preservatives to show the natural stability and colour development of a henna paste over the span of 8 weeks (Figure 1a,b). The gel formulation was prepared containing pH regulators as well as preservatives to mimic a more realistic cosmetic application. Both formulations were stored under light exclusion at 5°C after preparation.

The comparison of dyeing results for strands treated with the paste (Figure 1a,b) and those dyed with the gel containing aqueous henna extract (Figure 1c,d) resulted in a visibly different colour outcome. Strands dyed directly after paste formulation as well as on day 1 and day 7 after preparation exhibited a bright orange dye outcome highlighted by a*‐values between 27.8 and 30.9 as well as b*‐values of 47.5 and 47.9 (Table S1). A positive a*‐value corresponds to a red colour outcome and a positive b*‐value represents yellow [17]. The strands dyed with the gel showed a browner and more subdued colour compared to the paste (Figure 1a–d). Even though the gel preparation contained 0.36 ± 0.04% w/w lawsone, which is the maximum amount allowed by the SCCS when dyeing with a henna paste that contains ¼ henna powder with 1.4% w/w lawsone [7] resulting in 0.35% w/w of lawsone in the final dye mixture. The brown hue is represented by much lower a*‐ and b*‐values with 21.6 and 38.9 respectively for the dyed strands on day 1 after gel preparation (Table S1). The intensity and darkness of both colourations on day 1 are very similar, with L*‐values of 48.2 and 49.7 for the paste and the gel application respectively (Table S1).

The different colour outcomes could be due to a multitude of factors. Firstly, paste preparation is individual and can be influenced very easily. Depending on the temperature of the water used to prepare the paste, the lawsone release proceeds differently. Upwards of 85°C, the β‐glucosidase responsible for the hydrolysis of hennosides starts to denature, resulting in less lawsone being released and available for colouration. The cooling process is also dependent on the RT. The longer the paste is left out before applying to the hair, the more lawsone is released. The shown paste was prepared with 80°C water and was left to cool for 30 min. These parameters allowed the enzyme to act at higher temperatures without risking denaturation while still applying heat to speed up the extraction and hydrolysis process.

As a result of different extraction conditions, the lawsone‐rich extract (LE) contains more polyphenols than the paste. Since the ratio of solvent to plant powder for the LE extraction is 15 times higher than the ratio of solvent to plant powder when using a paste, proportionately more polyphenols can be extracted. In addition to the investigation of ideal conditions for lawsone extraction, Kavepour et al. [8] also evaluated the TPC content of their extracts, which displayed higher polyphenol contents in correlation to a higher solvent‐to‐raw material ratio. Joyroy et al. [18] investigated the total polyphenolic content (TPC) of different *Lawsonia inermis* L. extracts, finding that repeated extraction of ground henna leaves with boiling water led to an extract containing 12.47 ± 0.07% w/w of polyphenols. The longer extraction time of 2 h at 40°C in our work led to the extraction of 6.5 ± 0.07% w/w polyphenols in regard to the extract. The optimization of extraction conditions for polyphenols from *Lawsonia inermis* L. stems by Tan et al. [19] showed that after 270 min, the equilibrium of polyphenols in solution is reached and longer extraction times only led to a decrease in polyphenol yield, possibly due to degradation of extracted polyphenols in solution.

The presence of lawsone, polyphenols and 25 ppm of iron could lead to the formation of complexes between not only lawsone and metal [20] but also polyphenols and metal, as well as lawsone and polyphenols, leading to the colour shift towards brown. The multifaceted interaction of secondary metabolites with each other, as well as with metals present in the formulation, might be one of the reasons why the dye formulation did no longer colour the hair adequately after 4 weeks. Another reason for the lawsone degradation observed for LE extract in formulation could be the reaction of the orange dye with reducing sugars in the extract. The extract contains 21.1 ± 3.1% w/w reducing sugars, half of which is glucose. The high glucose levels result from the hydrolysis of hennosides during the extraction process, releasing glucose and hydrolawsone, which subsequently oxidizes to form lawsone. 2‐hydroxy‐1,4‐naphthoquinone has been proven to be a redox‐active compound [21, 22]. While current research predominantly focuses on the enzymatic reduction of naphthoquinones in oncological settings, it is highlighted that other reducing agents are also capable of spontaneous electron transfer, leading to the formation of highly reactive intermediates [21]. It could therefore be possible that lawsone is reduced in the gel, producing hydroquinones and semiquinones, causing the formation of reactive oxygen species (ROS) which could be the cause of an instable formulation that is not able to colour hair after 4 weeks. To draw definitive conclusions about the degradation of lawsone and the causal relationship with reducing sugars present in the system, further experimental investigations are required.

The colour outcome of strands dyed with LE gel 7 days after preparation was already significantly different to the colour outcome on day 0 (Figure 1c,d). The strands were lighter in colour and less brown, represented by the higher L* value and lower a*‐ and b*‐values (Table S1) and a Δ*E* value of 8.2 compared to the strands dyed on day 0. Especially, the dye results for strands coloured after 28 days as well as 56 days no longer compared to the colour results for day 0–7. The strands appeared ashy blond, with a Δ*E* value of 20.5 and 25.3 respectively compared to the strands dyed right after gel formulation (Figure 1c,d).

The paste preparation yielded consistent colour results until 21 days after preparation. Even though the Δ*E* values are significantly different to the strands dyed immediately after preparation, the Δ*E* values are only 3.2, 3.5 and 5.1 for colourations on day 1, 7 and 14, respectively, compared to day 0. Research has shown that a trained eye is able to differentiate colour with a Δ*E* of 1–2, whereas Δ*E* values of 2–10 are perceptible at a glance [23]. These studies do not include three‐dimensional specimens like hair strands, which, due to their physic, shine and varying dye uptake, will never be adequately described by just one set of L*‐, a*‐ and b*‐values. Therefore, strands with a Δ*E* value of approx. 3 are not necessarily distinguishable from strands dyed right after paste preparation. The difference in colour between the strands dyed at day 21 and day 0 was significant, with a Δ*E* of 9.5 because of higher L*‐values and lower a*‐values (Table S1). Especially, the comparatively high standard deviations of the strands dyed on day 21 and 28 showed that colour uptake became more inconsistent the longer the paste was stored before application. The decrease in colour intensity for the paste application can be explained by the composition of the paste. As mentioned, the paste was not preserved, enabling bacteria to grow during the span of 8 weeks. Even though there were no visible nor olfactorily perceivable changes, it is likely that natural bacteria present in the henna leaves were able to grow in the moist environment. Though it has been found that certain strains of *Pseudomonas* are able to degrade lawsone and other quinones [24, 25, 26] it is unlikely that these strands occured in the paste. More likely, lawsone was no longer available for colouration after week 4 due to its reactivity and affinity to proteinaceous material. Lawsone binds to protein via Michael addition [27]. With a lot of plant material present in the formulation, it is likely that the continuously released lawsone slowly bound to proteins in the paste and was therefore no longer available to covalently bind to the keratin during the dyeing procedure. Furthermore, it is possible that lawsone oxidized further and degraded over time due to lawsone's reported sensitivity to oxygen and UV‐light [8, 28]. Even though the paste was stored under light exclusion, it was removed for each dye application and was not stored under anaerobic conditions. The Δ*E* value of 24.2 for strands dyed after 8 weeks is comparable to the difference in colour for the gel application (Δ*E* 25.3).

Both application systems containing pre‐released lawsone do not yield consistent dye results and are therefore not suitable as alternatives for more consumer‐friendly plant‐based hair colouration products. When using a third dye application consisting of a two‐component system where the colour gel contains not lawsone but the naturally occurring hennosides and the second component containing a β‐glucosidase, one can observe a different trend (Figure 1e,f). Strands that were dyed with a mixture of an ethanolic extract (EE) in gel, which was combined with 50 μL of liquid β‐glucosidase per strand 15 min before application, showed a bright orange colouration throughout the 8 weeks of testing. The dyeing results over the span of 56 days did not display a Δ*E* value higher than 2.7 (day 14) compared to day 0, revealing no visible colour difference. The dye results were very comparable to the colour outcome of the paste on day 7 (Table S1) highlighting this formulation's suitability as an alternative to the paste application. Notably, the hennoside content in the EE gel formulation is 0.39% w/w, which, assuming 100% hydrolysis, corresponds to 0.20% w/w of lawsone. This is not only well below the allowable limit of 0.35%, but also lower than the amount found in the LE gel, which did not produce a vibrant colour payoff. Furthermore, the EE extract contained 22.7% w/w TPC and 22.3% w/w reducing sugars. Those components negatively affected the LE gel formulation, whereas they had no influence on the hennoside stability in gel as well as the dye outcome, emphasizing once more that hennosides are highly compatible ingredients for complex cosmetic formulations, as they are neither reactive nor unstable in formulation.

### Extraction optimization to separate hennoside and β‐glucosidase

**FIGURE 2:**
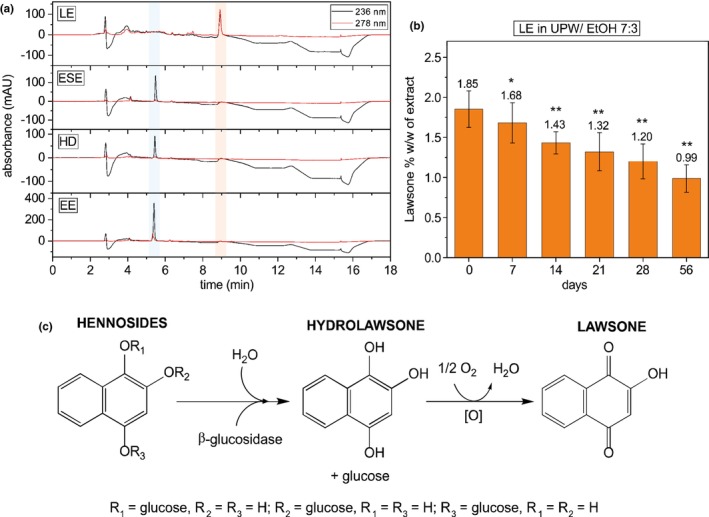
(a) HPLC profiles of a lawsone‐rich aqueous extract (LE, 100.17 μg mL^−1^), ethanolic Soxhlet extract (ESE, 7.8 μg mL^−1^), heat‐denatured aqueous extract (HD, 8.6 μg mL^−1^) and industrial‐scale ethanolic extract (EE, 11.2 μg mL^−1^), all dissolved in H₂O:EtOH (volume ratio 7:3). Absorbance at 236 nm (hennoside, black) and 278 nm (lawsone, red) over 18 min is shown; hennoside peaks are marked in blue, lawsone in orange; (b) Lawsone content (% w/w) in LE extract (H₂O:EtOH, 7:3) over 8 weeks. Significant differences from day 0: *p < 0.05, **p < 0.01; mean ± SD, *n* = 3; (c) Schematic of the enzymatic release mechanism: Three monoglycosidic hennosides hydrolysed by β‐glucosidase to hydrolawsone, which oxidizes to lawsone.

The investigation of lawsone‐rich extract in aqueous solution (volume ratio of 7:3 water to ethanol) showed that lawsone does indeed degrade over time even under light exclusion and without the influence of a complex gel formulation, pH regulators, and preservatives (Figure 2b). The amount of lawsone found after 7 days was already significantly lower at 1.68% w/w compared to 1.85% at day 0. After 14 days, the drop in lawsone concentration increased with 22.7% less orange dye detectable than on day 0. After 8 weeks, there was 0.99% w/w lawsone left, which is 53.5% of the lawsone amount originally present.

These findings underline the need to overthink the extraction and formula in which henna should be made available for consumers in the future. It is very well documented that lawsone only occurs in negligible amounts in the henna leaves but rather it is reduced and glycosylated precursors, called hennosides [6]. When ground up henna leaves are prepared in a paste, the addition of water initiates the enzymatic hydrolysis of said hennosides to form hydrolawsone and subsequently lawsone (Figure 2c). The leaves of the *Lawsonia inermis* L. shrub contain a β‐glucosidase that catalyses the mentioned hydrolysis as soon as water and leaves are combined [29].

First developments regarding glycosylated lawsone derivatives in cosmetic hair colouration formulations have been published by Wella [30]. The patent describes the potential inclusion of either a single lawsone glycoside or a combination of multiple glycosides in one formulation, alongside a second formulation containing a type of hydrolase. These formulations are to be combined shortly before application to generate orange to violet shades [30]. The concept of a controlled lawsone release is therefore already known and tested. What remains to be explored is how to effectively control the release of lawsone and how to optimize the extraction of henna raw material to produce hennoside‐rich extracts that lack hydrolase activity, particularly β‐glucosidase.

The aforementioned results have shown that the focus should be on the controlled release of lawsone shortly before application rather than on extracts containing readily available lawsone. Hennosides exhibit high water solubility attributable to their glucose moiety and demonstrate stability in aqueous formulations, given the absence of hydrolysing agents like enzymes or an extremely low pH. To date, no effective and scalable method has been published to isolate large amounts of hennoside from *Lawsonia inermis* L. material. Some studies have described the glycosides [29, 31], but the scalable isolation has yet to be investigated. Furthermore, it should be noted that isolating a single compound from plant material for use in cosmetic formulations is generally inefficient. This process generates large amounts of plant waste, consumes significant energy to carry out multiple purification steps and often involves the use of organic solvents to enhance product purity. Focusing on the production of a *Lawsonia inermis* L. extract rich in hennosides, along with other secondary metabolites, represents a practical compromise at the industrial scale.

There are several ways to extract henna raw material without triggering a lawsone release. At laboratory scale, we could show that ethanolic Soxhlet extraction (ESE) (Figure 2a, ESE) led to the extraction of 16.4 ± 2.8% w/w of hennosides in regard to the raw material (Table 2). The HPLC profile revealed the clear presence of hennoside as well as the absence of lawsone. Hennoside elutes at min 5.2, whereas lawsone is visible at min 8.7. The extraction with ethanol ensures the separation of β‐glucosidase and glycosylated dye precursors. The enzymes are neither soluble nor active in ethanol, resulting not only in the separation of β‐glucosidase and hennoside but also in the prevention of lawsone release. The inactivation of β‐glucosidase present in raw material can also be achieved by heat treatment before or during extraction. By extracting *Lawsonia inermis* L. raw material with 100°C boiling water, the β‐glucosidase is denatured, leading to the controlled extraction of hennosides (9.6 ± 2.4% w/w) without lawsone formation. HPLC analysis of the heat‐denatured extract (HD) illustrated the successful extraction of hennosides and the absence of lawsone (Figure 1a, HD). Without the ensured heat denaturation of β‐glucosidase, lawsone is released, as demonstrated by the HPLC profile of the lawsone‐rich extract (Figure 2a, LE). The extraction of raw material for 2 h at 40°C led to the complete hydrolysis of hennosides; the profile lacked a prominent peak at min 5.2, whereas there was a distinct peak at min 8.7, indicating the presence of lawsone.

Laboratory testing has shown that the extraction with ethanol (96%) yields higher amounts of hennoside compared to an aqueous extraction with boiling water. In addition, the aqueous extraction not only generated a viscous solution that was difficult to process but also posed challenges when scaled up since the boiling of large amounts of water is accompanied by high energy costs. Moreover, when working with larger quantities of raw material, achieving uniform and immediate contact between the henna and boiling water becomes logistically challenging. This necessitates a more advanced setup to ensure the successful translation of laboratory‐scale results to larger‐scale applications.

The first industrial scale extraction of 50 kg of *Lawsonia inermis* L. material (EE) yielded a water‐soluble extract with 29.7% w/w of hennosides and negligible amounts of lawsone, demonstrated by the HPLC profile (Figure 2a, EE). As described in detail under Industriale‐scale maceration targeting hennosides, the initial extract after maceration was processed with acetone and subsequently water to remove large amounts of chlorophyll and concentrate the hennoside in the extract. The processing allowed for a high performative extract that was easy to incorporate into a gel formulation. The downsides of the current protocol are the comparatively low extract yield with only 4% in regard to the raw material extracted, as well as the multitude of processing steps needed to generate the hennoside‐rich extract.

### Controlled lawsone release during hair colouration

In addition to significant amounts of hennoside, the plant extracts contained large amounts of reducing sugars as well as polyphenols and small amounts of iron (Table 2). In the case of heat‐denatured extract (HD) the presence of amino acids as well as peptides and certain proteins is likely as well. As already established, interactions between phenolic substances and metals can influence not only the colour stability but also its shade and longevity. Redox reactions between polyphenolic structures and reducing sugars can impact the extract composition as well. The extracts themselves, as products of a natural source, are complex systems, highlighting that hennoside content alone does not give sufficient information about the colouring potential and ability of the extract at hand. With yak hair as a third reaction partner in a dye application, the potential interaction of other extract compounds with the hair fibre has to be considered.

The dye results for the ethanolic Soxhlet extract (ESE) show that the extract alone barely coloured the hair strand (Figure 2a,b). The Δ*E* value of 10.8 indicated a difference in colour compared to the control strand, primarily due to the slight yellow shift highlighted by the higher b*‐value of 16.7 compared to 9.8 for the control strand (Table S2). The slight tint could be due to polyphenols attaching to the hair fibre. It has been reported that henna does contain quercetin, kaempferol, myricetin, luteolin and small amounts of certain aurones which are known to be yellow [32]. There was no characteristic orange colouration since lawsone was not present in the extract and hennosides are neither orange nor do they bind to the hair fibre in their glycosylated state. This result confirms the need for a second component to initiate the hydrolysis of isolated hennosides to form lawsone shortly before application.

**FIGURE 3:**
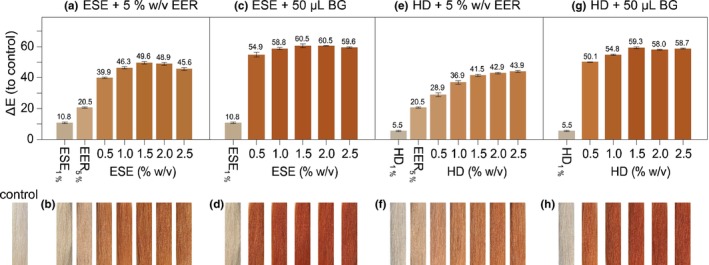
(a, c, e, g) Mean ± SD of ∆*E* values for hair strands dyed with ethanolic Soxhlet extract (ESE) or heat‐denatured aqueous extract (HD), either with 50 μL β‐glucosidase per strand or with 5 w/v % EER each in 25 mL ultra‐pure water per strand, compared to undyed control strands, (*n* = 3); (b, d, f, h) Corresponding images of dyed strands shown beneath each ∆*E* column, Column colours reflect CIELab values converted to RGB based on Lab‐means.

As previously mentioned, the henna β‐glucosidase is not soluble in ethanol and therefore stays in the plant material that is extracted. The ethanolic extraction temporarily inhibits the enzyme activity but does not actively denature the enzyme. When combining different concentrations of ESE with 5% w/v of the extracted residue material (ethanolic extraction residue, EER) an orange colour formation was visible (Figure 3a,b). The colouration with 5% w/v EER alone showed only a very light orange tint, implying that almost all hennoside was extracted and is no longer available for the enzyme to hydrolyse and form lawsone. The most intense colouration was achieved by combining 1.5% w/v ESE and 5% w/v EER 15 min before dyeing the hair for 30 min in suspension. These dyeing results confirm that the separation of hennoside and enzyme from the same material is possible as well as the initiation of the controlled lawsone release by recombining the products of the extraction.

The colour results when using EER as the catalyst are significantly less intense than the results achieved by combining different concentrations of ESE with 50 μL of liquid beta‐glucosidase per strand (Figure 3c,d). This is due to a multitude of factors. Firstly, 5% w/v of EER in suspension complicated the diffusion. The excessive plant material in suspension obstructed the contact of released lawsone with the hair fibre and led to an uneven colour result. Secondly, preliminary tests in our work have shown that the henna β‐glucosidase is difficult to extract from the plant material. The natural pH of a henna suspension (5% w/v) as well as the pH of 1.5% w/v ESE and 5% w/v EER in suspension lies between 4.0 and 4.5. The glucosidase is more soluble in alkaline conditions. Therefore, only a small amount of enzyme is available in solution to hydrolyse the dissolved hennoside, explaining the comparatively light orange dye outcome.

The colourations with the liquid beta‐glucosidase product as the catalyst resulted in vibrantly coloured strands depicting a Δ*E* value of 58.8 (1% ESE) and 60.5 (1.5% ESE) compared to the control strand. The dyeing results for the heat‐denatured aqueous extract (HD) in combination with EER showed a similar trend to the colourations of ESE and EER. HD alone did not colour the hair and lacked the yellow tint to the hair fibre noticeable for ESE. This can be explained by the different solvents used for ESE and HD. Even though the TPC contents of both extracts are comparable, with 10.7 ± 0.49% w/w for ESE and 9.9 ± 0.80% w/w for HD (Table 2) the responsible polyphenols for the yellow hue were primarily extracted with ethanol and did not show on the hair after HD colouration. The combination of HD and EER led to orange coloured hair; though the colouration lacked intensity, the best colour outcome with 2.5% w/v HD and 5% w/v EER resulted in a Δ*E* value of 43.9, with a L*‐value of 57.6 and an a*‐value of 22.3. These results are very different from the best colour result when combining HD with the liquid β‐glucosidase sample. The colouration of 1.5% w/v HD resulted in a Δ*E* value of 59.3, with an L*‐value of 46.0 and an a*‐value of 33.5, which represents a much darker and redder hair strand.

Concentrations between 1% w/v and 1.5% w/v for ESE and HD are enough in combination with the liquid enzyme sample to generate intensely orange coloured hair strands. Even though the compositions of the extracts are different, the colour outcomes are comparable. The only differences are the slightly higher a*‐values for ESE colourations compared to the HD applications (Table S2).

These results show that the composition of the extract can have immense impact on the stability of the extract in formulation, but in regard to the colouration of the hair, hennoside content is the most important marker. A dye application in solution with 1% w/v ESE is comparable to a colouration with 1.5% w/v HD; the amount of hennoside available in solution is 41 ± 7 mg per strand for ESE and 36 ± 9 mg for HD respectively. Those amounts are equivalent to 21.1 ± 3.6 mg and 18.6 ± 4.6 mg lawsone per strand respectively. In the dye systems at hand, a 100% hydrolysis would result in a concentration of 0.084 ± 0.014% w/v for ESE and 0.074 ± 0.018% w/v for HD. These amounts are substantially lower than the 0.35% that could be released during a paste application. Using a hennoside‐rich extract in solution or a gel formulation in combination with a liquid β‐glucosidase as a catalyst not only results in vibrant colours but shows that the targeted hydrolysis of hennosides enables the usage of much less source material, which in turn means that the consumer is exposed to lower levels of lawsone in comparison to a paste application.

### Washing fastness of different henna applications

Due to lawsone's ability to bind covalently to the hair fibre [27], henna colouration has a higher washing fastness compared to other plant‐based colouration, which heavily rely on temporary interactions of dye molecules and keratin fibre, like anthocyanins from black beans, which withstand only 4 washes [33]. By incorporating an ethanolic extract produced at industrial scale (EE) into a gel formulation and forcing the lawsone release by the addition of a liquid β‐glucosidase shortly before application, we could achieve a comparable colouration to a traditional paste. To make a final statement regarding the suitability of a hennoside‐based gel application as an alternative to the traditional application, the washing fastness compared to that of the paste is of great importance.

**FIGURE 4:**
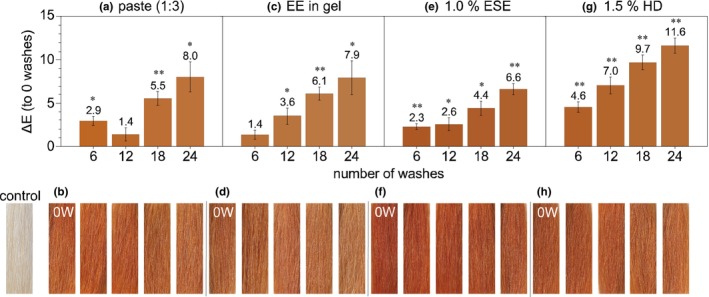
(a, c, e, g) Mean ± SD of ∆*E* values for hair strands dyed with henna paste, ethanolic gel extract (EE), ethanolic Soxhlet extract (ESE), or heat‐denatured extract (HD), each washed 6–24 times and compared to unwashed strands (0 W), *n* = 3; (b, d, f, h) Corresponding strand images for 0–24 washes, shown below each column; Column colours represent lab‐means translated to RGB. Significant differences from unwashed strands are indicated by *p < 0.05, **p < 0.01.

The Δ*E* after 12 washes for strands coloured with the paste was 1.4 and statistically not significantly different to the Δ*E* of colourations before washing. As mentioned before, the colouration of hair as a complex and three‐dimensional matrix results in a colour outcome that can vary depending on light position and colour uptake by individual hairs. Even though the Δ*E* after 6 washes amounted to 2.9, there was no visible difference between strands before washing, after 6 and 12 washes (Figure 4a,b). The average L*‐values for all three stayed around 53, proving that the strands did not become lighter after 12 washes. Solely the a*‐ and b*‐values decreased slightly, resulting in a less intense but not lighter colour after 12 washes (Table S3). After 18 and 24 washes, the strand did increase in lightness. The orange colour was more subdued and appeared browner (Figure 4a,b). Even though the Δ*E* of 8 represents a significant and noticeable colour shift, the hair strands are still strongly coloured and resemble the strands before washing more than the control strand. The washing fastness for EE in gel is comparable to the paste. The strands did not differ in colour after 6 washes; the Δ*E* of 1.4 was not significantly different to the colouration before the first washing cycle. The lightness increased slightly after 12 washes, from an L*‐value of 53.7 to 55.6. After 18 and 24 washes, the strands did appear significantly lighter in colour, but compared to the paste application, there was no colour shift to brown noticeable. The colour faded very evenly while keeping the same hue over the span of 24 washes (Figure 4c,d).

The washing fastness of strands dyed in solution with the ethanolic Soxhlet extract (ESE) was the strongest compared to all colourations. The Δ*E* after 24 washes only amounted to 6.6, with an increase in lightness of just 4.3 and a lower a*‐value by 4.8 (Table S3). Even if the colour outcome is mainly influenced by the hennoside and therefore released lawsone present in solution, the addition of polyphenols could have a positive impact on the colour stability. Possible complexes of lawsone with polyphenols could lead to the increased durability of the colour. A recent in vitro study has found that quercetin and rutin, two polyphenols present in henna, have a stabilizing effect on blue colouration of anthocyanins in honeysuckle juice [34]. The reported non‐covalent complexes between anthocyanins and polyphenols could also be established between naphthoquinones like lawsone and polyphenols on the hair fibre, shielding the lawsone not only from further oxidation but also positively affecting lawsone's washing resistance. With lawsone covalently bound to the hair fibre, this hypothesis needs to be investigated further to proof that non‐covalent interactions of functional groups between polyphenols and lawsone are possible as well.

The dye results of 1.0% ESE and 1.5% HD were comparable (Figure 3e,g) but the washing stability of these colourations differed (Figure 4e–h). Colourations with 1.5% HD in solution showed a worse washing fastness compared to all application forms even though the initial dye outcome was very intense and darker than the paste and gel applications. Six washes resulted in a Δ*E* of 4.6 with a significant increase in lightness, by a difference in L*‐value of 3.9 which is comparable to the lightening of strands dyed with ESE after 24 washes. Strands dyed with HD faded evenly throughout the washing process but lost colour intensity early into the washing fastness test. After 24 washes, the strands were significantly lighter and less intense in orange colouration. The Δ*E* of 11.6 represents the largest difference of strands after 24 washes compared to the undyed strands out of all application methods. As mentioned before, the composition of extracts could positively influence the colour stability in the case of an increased polyphenol content for ESE. With HD, the significant changes after six washes could indicate the presence of non‐covalently bound lawsone on the hair fibre that is easily removed after a few washes. This could be due to other components in the extract that interact with the hair fibre during the colouration process and block some of the released lawsone from covalently binding to the keratin. These results are of interest for the future optimization of the extract and need to be investigated further to improve the extract composition for formulation purposes.

In summary, we demonstrated that the reinvention of henna‐based hair colouration in a convenient formulation is feasible by effectively controlling the lawsone release mechanism. This finding provides a solid foundation for the development of a performant, stable and plant‐based hair dye that offers several advantages over traditional paste‐based applications. The two‐component system mirrors the format familiar to consumers from oxidative hair dyes, ensuring a smooth and straightforward application process, including rinsing. The light gel texture allows for easy distribution, resulting in an even colour outcome with wash fastness comparable to that of the paste. By offering plant‐based hair colouration in a more user‐friendly format, the barrier to consumer engagement is addressed. Consequently, messy, time‐consuming and difficult‐to‐control paste applications would no longer be the sole effective option for sustainable, plant‐derived hair colouring.

A hennoside‐rich extract produced at industrial scale in combination with a β‐glucosidase proved to be a promising base for future product development. Preliminary storage tests have shown that both the extract in formulation as well as the β‐glucosidase in a simple gel formulation packaged in aluminium tubes are stable over the time of 12 weeks at 40°C. Assessment of changes in pH, viscosity, changes of the packaging material as well as weekly performance tests of the formulations have shown promising results, indicating the suitability of the shown formulation as a platform for future product development.

## CONCLUSION

The formulation of henna extracts into consumer‐friendly applications revealed a number of challenges for product development. Lawsone, as a dye, is not only highly regulated and poorly soluble in water, but also unstable due to UV‐sensitivity and its redox potential. Application formulations working with readily available lawsone do not perform consistently and fail to colour yak hair beginning 4 weeks after formulation. Incorporating naturally occurring mono‐glycosides of lawsone into the formulation led to a stable application that could be combined with a β‐glucosidase before hair colouration to release lawsone. By controlling the release mechanism, it was ensured that lawsone was released shortly before dyeing the hair, therefore preventing the degradation of lawsone and allowing for vibrant colouration even after 8 weeks of storage.

Furthermore, several extracts were compared regarding their dyeing abilities as well as washing fastness to assess their suitability and scalability for future extraction and formulation optimizations. An ethanolic extract produced at industrial scale was found to be the most promising for subsequent product optimization since the colouration outcome as well as the washing fastness compared to the traditional paste application.

## CONFLICT OF INTEREST STATEMENT

The authors declare that they have no conflicts of interest regarding the publication of this article.

## Supporting information


**Table S1.** Average and standard deviation (SD) of L*‐, a*‐ and b*‐values for colouration results for a paste (1:3 weight volume of plant material to water) a gel formulation containing lawsone‐rich henna extract (LE) and a gel formulation containing ethanolic henna extract (EE) that have been stored and used for colouration over the span of 8 weeks, *n* = 5 strands for each colouration respectively.
**Table S2.** Average and standard deviation (SD) of L*‐, a*‐ and b*‐values for colouration results of ethanolic Soxhlet extract (ESE), ethanolic Soxhlet residue material (EER), heat‐denatured aqueous henna extract (HD) and liquid β‐glucosidase fermentation product in combination at different concentrations (% w/w), *n* = 3 strands for all colourations respectively.
**Table S3.** Average and standard deviation (SD) of L*‐, a*‐ and b*‐values for washing fastness results of colourations with a paste (1:3 weight volume of plant material to water, 5 g per strand), a gel formulation containing ethanolic henna extract (EE, 5 g gel + 50 μL β‐glucosidase per Strand), colourations in solution containing ethanolic Soxhlet extract (ESE, 1% w/v, 50 μL β‐glucosidase per strand) and heat‐denatured aqueous extract (HD, 1.5% w/v, 50 μL β‐glucosidase per strand).

## Data Availability

The data that support the findings of this study are available from the corresponding author upon reasonable request.
