# Supplementary material for: Reinventing henna: Enzyme‐catalysed colour release from stabilized Lawsonia inermis L. extracts
Source: Int J Cosmet Sci. 2025 Oct 20;48(2):310–26. doi: 10.1111/ics.70029 (PMC13068042; doi:10.1111/ics.70029)
Supplement: Supplementary file 1 — Table S1. Average and standard deviation (SD) of L*‐, a*‐ and b*‐values for colouration results for a paste (1:3 weight volume of plant material to water) a gel formulation containing lawsone‐rich henna extract (LE) and a gel formulation containing ethanolic henna extract (EE) that have been stored and used for colouration over the span of 8 weeks, n = 5 strands for each colouration respectively. Table S2. Average and standard deviation (SD) of L*‐, a*‐ and b*‐values for colouration results of ethanolic Soxhlet extract (ESE), ethanolic Soxhlet residue material (EER), heat‐denatured aqueous henna extract (HD) and liquid β‐glucosidase fermentation product in combination at different concentrations (% w/w), n = 3 strands for all colourations respectively. Table S3. Average and standard deviation (SD) of L*‐, a*‐ and b*‐values for washing fastness results of colourations with a paste (1:3 weight volume of plant material to water, 5 g per strand), a gel formulation containing ethanolic henna extract (EE, 5 g gel + 50 μL β‐glucosidase per Strand), colourations in solution containing ethanolic Soxhlet extract (ESE, 1% w/v, 50 μL β‐glucosidase per strand) and heat‐denatured aqueous extract (HD, 1.5% w/v, 50 μL β‐glucosidase per strand). [file ICS-48-310-s001.docx]

**Supporting Information – Reinventing Henna: Enzyme-Catalysed Colour Release from Stabilized *Lawsonia inermis* L. Extracts**

**Running Title: Henna hair dye – an improved two-component system**

Authors: Nele Dallmann^1,2^, Volkmar Vill^2^, Fabian Straske^1,2^

nele.dallmann@uni-hamburg.de, Phone: +49 40 88242116

volkmar.vill@uni-hamburg.de, Phone: [49 40 42838-4269](tel:+49-40-42838-4269)

fabian.straske@henkel.com, Phone: +49 40 88242036

^1^Henkel AG & Co. KGaA, Ruhrstraße 19, 22761 Hamburg, Germany

^2^University of Hamburg, Department of Organic Chemistry, Martin-Luther-King-Platz 6, 20146 Hamburg, Germany

Correspondence: Fabian Straske, Henkel AG & Co. KGaA, Ruhrstraße 19, 22761 Hamburg, Germany, E-mail: fabian.straske@henkel.com, Phone: +49 40 88242036

**Table S1** Average and standard deviation (SD) of L*-, a*- and b*-values for colouration results for a paste (1:3 weight volume of plant material to water) a gel formulation containing lawsone-rich henna extract (LE) and a gel formulation containing ethanolic henna extract (EE) that have been stored and used for colouration over the span of 8 weeks, n=5 strands for each colouration respectively.

| **Dye Formulation** | **Day** | **L*±SD** | **a*±SD** | **b*±SD** |
| --- | --- | --- | --- | --- |
| paste | 1 | 48.2±0.4 | 3096±0.9 | 47.9±1.4 |
|  | 7 | 51.9±0.5 | 27.8±0.9 | 47.5±0.9 |
|  | 14 | 53.6±0.4 | 26.5±1.0 | 47.6±1.1 |
|  | 21 | 55.4±1.2 | 23.2±2.0 | 45.1±2.4 |
|  | 28 | 54.8±2.5 | 21.7±3.4 | 42.5±3.8 |
|  | 56 | 61.9±1.2 | 14.2±1.2 | 35.3±1.5 |
| LE in gel | 1 | 49.7±0.7 | 21.6±0.9 | 38.9±1.2 |
|  | 7 | 51.9±1.7 | 18.8±0.9 | 35.4±0.9 |
|  | 14 | 53.6±0.4 | 17.1±0.4 | 32.7±0.4 |
|  | 21 | 52.2±0.4 | 15.2±0.1 | 30.5±0.3 |
|  | 28 | 54.8±2.5 | 21.7±3.4 | 42.5±3.8 |
|  | 56 | 57.6±1.2 | 9.8±0.4 | 21.4±0.9 |
| EE in gel | 1 | 51.2±0.4 | 26.7±0.8 | 43.6±1.2 |
|  | 7 | 52.8±1.2 | 26.9±0.6 | 43.3±1.1 |
|  | 14 | 52.9±1.3 | 27.7±1.2 | 44.5±0.7 |
|  | 21 | 53.0±1.0 | 27.2±0.8 | 44.8±0.9 |
|  | 28 | 52.4±1.4 | 27.2±1.4 | 44.4±1.4 |
|  | 56 | 53.1±0.6 | 26.5±0.9 | 44.7±0.7 |

**Table S2** Average and standard deviation (SD) of L*-, a*- and b*-values for colouration results of ethanolic Soxhlet extract (ESE), ethanolic Soxhlet residue material (EER), heat denatured aqueous henna extract (HD) and liquid β-betaglucosidase fermentation product in combination at different concentrations (% w/w), n=3 strands for all colourations respectively.

| **Dye Formulation** | **Conc. % w/v** | **L*±SD** | **a*±SD** | **b*±SD** |
| --- | --- | --- | --- | --- |
| Control undyed | **-** | 78.3±0.2 | 0.1±0.1 | 9.8±0.2 |
| ESE control | 1.0 | 70.1±0.5 | 3.8±0.2 | 16.7±0.5 |
| EER | 5.0 | 70.0±0.3 | 9.5±0.3 | 26.1±0.4 |
| HD | 1.0 | 73.4±0.3 | 2.0±0.1 | 11.6±0.4 |
| ESE + 5 % EER | 0.5 | 59.4±0.2 | 18.6±0.2 | 39.6±0.3 |
|  | 1.0 | 55.3±0.1 | 22.4±0.7 | 43.2±0.5 |
|  | 1.5 | 52.2±0.4 | 24.0±0.9 | 44.6±0.9 |
|  | 2.0 | 52.5±0.4 | 23.4±0.7 | 44.2±0.7 |
|  | 2.5 | 54.4±0.2 | 21.1±0.8 | 42.4±0.8 |
| ESE + 50 µL BG | 0.5 | 49.8±1.1 | 30.4±1.5 | 45.6±0.3 |
|  | 1.0 | 46.2±0.9 | 33.9±0.4 | 45.6±0.1 |
|  | 1.5 | 45.9±0.5 | 35.4±0.5 | 46.8±1.2 |
|  | 2.0 | 46.0±0.9 | 35.4±0.2 | 46.9±0.9 |
|  | 2.5 | 45.9±0.4 | 34.6±0.3 | 46.0±0.7 |
| HD + 5 % EER | 0.5 | 65.3±0.6 | 13.8±0.7 | 31.7±1.4 |
|  | 1.0 | 61.8±0.3 | 18.7±0.5 | 37.0±1.2 |
|  | 1.5 | 59.5±0.5 | 20.9±0.2 | 40.4±0.5 |
|  | 2.0 | 58.7±0.7 | 21.8±0.3 | 41.2±0.2 |
|  | 2.5 | 57.6±0.3 | 22.3±0.5 | 41.5±0.7 |
| HD + 50 µL BG | 0.5 | 56.6±0.2 | 26.7±0.1 | 46.2±0.4 |
|  | 1.0 | 49.6±0.6 | 29.4±0.3 | 46.2±0.1 |
|  | 1.5 | 46.0±0.2 | 33.5±0.4 | 46.7±0.8 |
|  | 2.0 | 47.1±0.7 | 32.8±0.2 | 46.2±0.6 |
|  | 2.5 | 47.5±0.1 | 33.0±0.4 | 47.5±0.6 |

**Table S3** Average and standard deviation (SD) of L*-, a*- and b*-values for washing fastness results of colourations with a paste (1:3 weight volume of plant material to water, 5 g per strand), a gel formulation containing ethanolic henna extract (EE, 5 g gel + 50 µL β-glucosidase per Strand), colourations in solution containing ethanolic Soxhlet extract (ESE, 1 % w/v, 50 µL β-glucosidase per strand) and heat denatured aqueous extract (HD, 1.5 % w/v, 50 µL β-glucosidase per strand).

| **Dye Formulation** | **washes** | **L*±SD** | **a*±SD** | **b*±SD** |
| --- | --- | --- | --- | --- |
| paste | 0 | 53.4±1.4 | 30.4±1.2 | 50.2±1.2 |
|  | 6 | 53.0±0.2 | 32.7±0.5 | 52.0±0.5 |
|  | 12 | 53.2±1.0 | 30.6±0.9 | 48.8±0.8 |
|  | 18 | 54.8±0.3 | 27.6±0.4 | 45.6±0.9 |
|  | 24 | 55.8±0.9 | 25.6±1.1 | 44.2±1.1 |
| EE in gel | 0 | 53.7±0.6 | 26.2±0.8 | 43.4±1.1 |
|  | 6 | 54.7±0.4 | 25.3±0.4 | 43.6±0.4 |
|  | 12 | 55.6±0.9 | 24.0±0.6 | 41.4±0.3 |
|  | 18 | 57.2±1.5 | 22.4±0.4 | 40.1±0.7 |
|  | 24 | 57.0±0.2 | 20.9±1.7 | 38.6±1.2 |
| ESE | 0 | 45.2±0.1 | 34.1±0.5 | 44.6±0.4 |
|  | 6 | 47.1±0.2 | 33.1±0.3 | 45.3±0.6 |
|  | 12 | 47.1±0.7 | 32.7±0.4 | 45.5±0.9 |
|  | 18 | 48.5±0.9 | 31.1±0.5 | 44.4±0.5 |
|  | 24 | 49.5±0.9 | 29.3±0.4 | 43.1±0.7 |
| HD | 0 | 46.0±0.2 | 33.5±0.4 | 46.7±0.8 |
|  | 6 | 49.9±0.7 | 31.2±0.4 | 47.0±1.3 |
|  | 12 | 51.5±0.9 | 29.1±0.6 | 46.4±0.7 |
|  | 18 | 52.8±0.8 | 26.9±0.8 | 44.5±1.0 |
|  | 24 | 53.6±0.8 | 25.2±0.6 | 43.6±0.9 |
